# Clinical Outcomes of Group D Retinoblastoma at a Tertiary Care Hospital in Pakistan

**DOI:** 10.7759/cureus.92151

**Published:** 2025-09-12

**Authors:** Khawaja Muhammad Ammar Ali Javed, Usman Vayani, Anum Javed, Muhammad Hanif Chatni

**Affiliations:** 1 Medicine, University College London, London, GBR; 2 Ophthalmology, University of Birmingham, Birmingham, GBR; 3 Ophthalmology, Patel Hospital, Karachi, PAK; 4 Ophthalmology, Al-Ibrahim Eye Hospital, Karachi, PAK

**Keywords:** cryotherapy, globe salvage rates, group d retinoblastoma, intravenous chemotherapy, intravitreal melphalan, laser therapy, low- and middle-income countries, multidisciplinary care, pediatric oncology, resource-limited settings

## Abstract

Purpose

To evaluate the clinical outcomes of Group D retinoblastoma (RB) managed with intravenous chemotherapy (IVC) and local therapies at a tertiary care center in Pakistan over nine years, with a focus on globe salvage rates and their implications for resource-limited settings.

Methods

This retrospective, cross-sectional study included patients diagnosed with Group D RB, classified under the International Classification of Retinoblastoma (ICRB), at Patel Hospital, Karachi, from April 2013 to December 2022. Data were collected on demographics, presenting symptoms, laterality, treatment protocols, and outcomes. Tumor evaluation was conducted under general anesthesia using indirect ophthalmoscopy. The primary treatment involved IVC with vincristine, etoposide, and carboplatin, supplemented by local therapies such as laser and cryotherapy. Intravitreal melphalan was administered in select cases with persistent vitreous seeds. Kaplan-Meier survival analysis was used to estimate globe salvage rates at one, two, and three years.

Results

Out of 170 patients with RB, 19 were identified with Group D disease, and 15 underwent globe salvage attempts. The median age at diagnosis was 32 months, and bilateral disease was predominant (93%). Leukocoria (60%) and strabismus (20%) were the most common presenting signs. Globe salvage was achieved in 73.33% (11/15) of eyes, with a mean follow-up duration of 57.5 weeks (range: seven to 263 weeks). Kaplan-Meier survival rates demonstrated overall globe salvage rates of 93%, 76%, and 65% at one, two, and three years, respectively. Four eyes of four patients underwent secondary enucleation due to tumor recurrence, retinal detachment, or persistent vitreous seeds, with histopathological findings of high-risk features in two cases. Importantly, no cases of metastasis or mortality were reported during the study period.

Conclusion

This study highlights the feasibility and effectiveness of IVC coupled with local therapies in achieving high globe salvage rates in Group D RB, even in resource-limited settings. Despite the challenges of advanced disease presentation and socioeconomic barriers, these results underscore the importance of multidisciplinary care and the need to expand access to specialized treatment centers in low- and middle-income countries (LMICs). Future efforts should focus on early diagnosis and the implementation of standardized treatment protocols to improve outcomes for advanced RB.

## Introduction

Retinoblastoma (RB) is the most common intraocular malignancy in children, with a global incidence of approximately one in 18,000 live births [1]. In Karachi, Pakistan, the annual crude incidence has been reported as 4.0 per 100,000 in children under five years of age and 2.4 per 100,000 in children under ten years [2]. RB is potentially curable; however, successful treatment depends on early detection and prevention of disease progression. The global mean age at presentation is 30.5 months, with a significant disparity between high-income countries and low- to middle-income countries (LMICs), including Pakistan [3]. Delayed presentation in LMICs contributes to a higher disease burden, as patients are more likely to present with advanced stages that are more difficult to treat [4].

Several classification systems have been proposed over time, with the International Classification of Retinoblastoma (ICRB) currently in use in Pakistan. This system primarily considers the extent of vitreous and subretinal seeding, in addition to tumor size and location [5]. Eyes are categorized into Groups A through E. Group D eyes are defined by diffuse vitreous or subretinal seeding and/or large, poorly circumscribed endophytic or exophytic tumors that have not yet progressed to Group E [6].

Salvaging Group D eyes is particularly challenging and remains an area of ongoing debate among ophthalmologists and pediatric oncologists. Group D lies between Groups C and E, while Group C eyes are often salvageable, Group E typically requires upfront enucleation. Management of Group D usually involves intravenous chemotherapy (IVC) in combination with local consolidative treatments such as laser photocoagulation and cryotherapy. Enucleation is performed when local consolidation fails. Reported globe salvage rates for Group D eyes with IVC alone range from 11% to 47%, whereas newer approaches such as intra-arterial chemotherapy have demonstrated salvage rates of approximately 45% [7].

This study presents the clinical outcomes of patients with Group D RB treated at a tertiary care hospital in Karachi, Pakistan. These findings may help predict globe salvage rates in this population and provide valuable prognostic information for tertiary centers in developing countries that adopt a multidisciplinary approach. This article was previously posted as a Research Square preprint: Javed KM, Vayani U, Javed A, Chatni MH. Clinical Outcomes of Group D Retinoblastoma at a Tertiary Care Hospital in Pakistan; April 06, 2023.

## Materials and methods

Study design

This retrospective, cross-sectional study was conducted at Patel Hospital, Karachi, Pakistan, spanning nine years, from April 2013 to December 2022. The study focused on evaluating the clinical outcomes of Group D RB treated at the institution.

Inclusion and exclusion criteria

Patients with a diagnosis of intraocular Group D RB based on the ICRB were included in the study. Both unilateral and bilateral cases were considered if at least one eye fulfilled the Group D criteria. Exclusion criteria included patients with incomplete medical records, less than three months of follow-up, or a diagnosis other than Group D RB.

Data collection

Data were extracted from patient records and included demographic variables (age, sex, family history), presenting symptoms (leukocoria, strabismus, proptosis), tumor laterality (unilateral or bilateral), and tumor characteristics such as the extent of vitreous and subretinal seeding. Tumor evaluation was performed using indirect ophthalmoscopy via examination under anesthesia (EUA). Follow-up data on treatment response, complications, and outcomes were also recorded. Histopathological reports following enucleation were reviewed for high-risk features.

Treatment protocols

Patients underwent a multimodal treatment approach consisting of systemic IVC combined with local consolidative therapies in the form of laser photocoagulation and cryotherapy, applied at the discretion of the treating ophthalmologist. Intravitreal melphalan (IViC) was considered in cases resistant to IVC and local therapy. Enucleation decisions were made at multidisciplinary tumor board meetings. Adjuvant chemotherapy was given when high-risk histopathological features were identified.

Systemic chemotherapy

All patients received the standard vincristine, etoposide, and carboplatin regimen. Each cycle included vincristine (1.5 mg/m² IV), etoposide (150 mg/m² IV), and carboplatin (560 mg/m² IV), administered every three to four weeks. The number of cycles ranged between six and eight, based on treatment response and disease progression.

Local therapies

Laser photocoagulation was delivered using an argon laser to consolidate tumors and reduce residual tumor burden. It was performed during EUA sessions.

Cryotherapy was applied to tumors in peripheral retinal areas, particularly in cases with significant subretinal seeding.

Intravitreal melphalan was administered in select cases with persistent vitreous seeds refractory to other treatments. The dose was 20-30 µg injected directly into the vitreous under sterile conditions.

Follow-up assessments

Patients were monitored via EUA at regular intervals. Assessments included indirect ophthalmoscopy, fundus photography, and ultrasonography to evaluate tumor response and complications. Follow-up frequency was every four to six weeks during chemotherapy and subsequently every eight to 12 weeks post-treatment. The mean follow-up duration was 57.5 weeks (range: seven to 263 weeks).

Outcome measures

The primary outcome was globe salvage, defined as retention of the affected eye without enucleation. Secondary outcomes included treatment-related complications (e.g., retinal detachment, persistent vitreous seeds), metastasis, and mortality. Globe salvage rates were calculated at one, two, and three years post-treatment.

Statistical analysis

Data were analyzed using IBM SPSS Statistics for Windows, Version 26 (Released 2019; IBM Corp., Armonk, New York, United States). Continuous variables were presented as means and ranges, while categorical variables were reported as percentages. Kaplan-Meier survival curves were used to estimate globe salvage rates over time. Log-rank tests were performed to compare survival curves where applicable.

Ethical considerations

This study was approved by the institutional review board at the Patel Hospital, Karachi. All patient data were de-identified prior to analysis, and confidentiality was maintained in accordance with the institutional policies.

## Results

**Figure 1:**
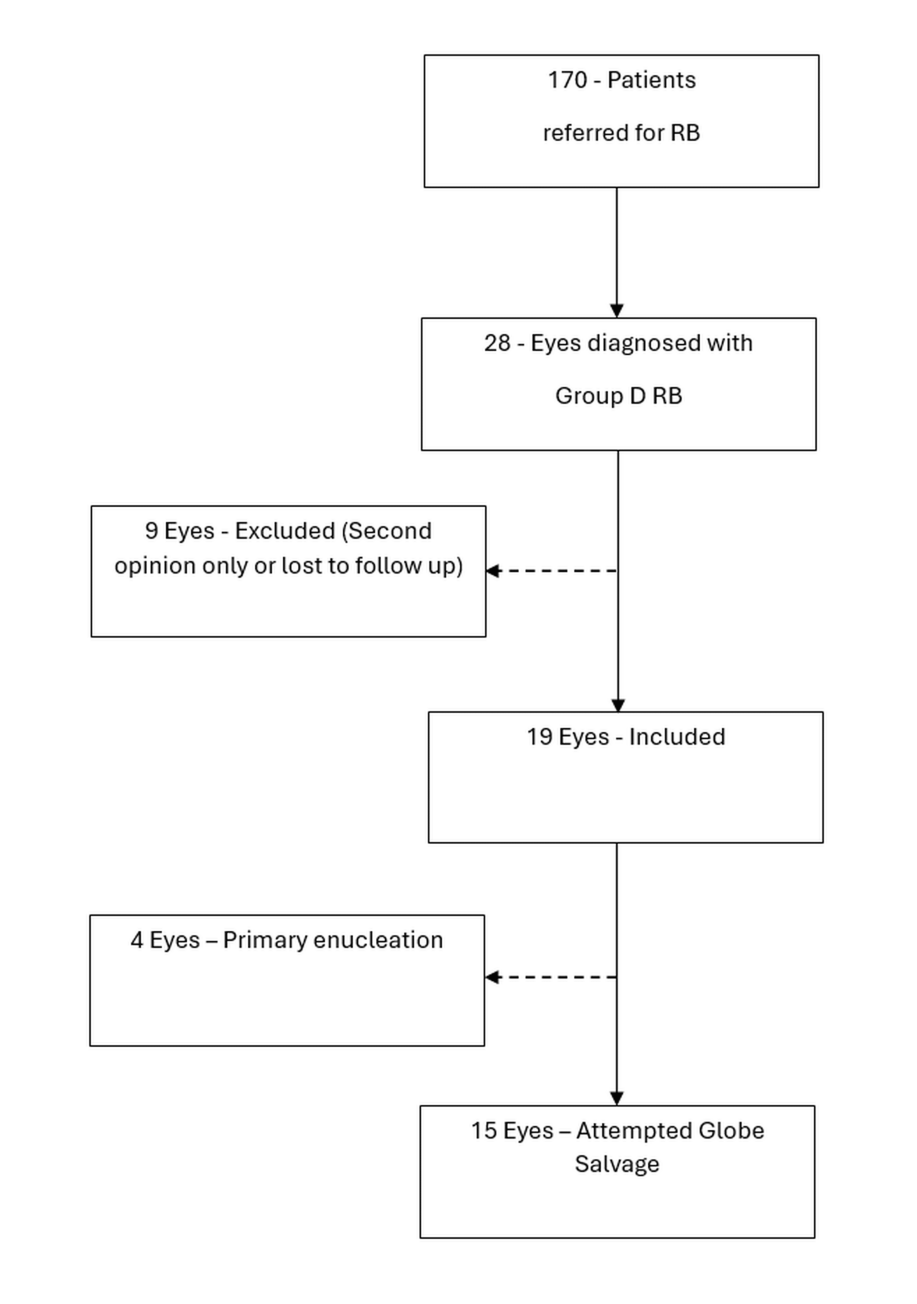
Flowchart showing patient selection RB: Retinoblastoma; One hundred and seventy patients referred, 28 eyes diagnosed with Group D, nine excluded (second opinion only or lost to follow-up), 19 eyes included in study, with four eyes undergoing primary enucleation and 15 eyes attempting globe salvage.

Between 25th April 2013 and 1st December 2022, 170 patients were referred to the Ophthalmology department at the Patel Hospital for RB. Group D RB was diagnosed in 28 patients during EUA. Of these, 22 patients had bilateral disease with Group D in at least one eye, one had bilateral Group D disease, whereas six had unilateral Group D disease. Nine patients were excluded from the study because they were examined either for second opinion only or there was loss of follow-up. Therefore, 19 Group D eyes of 19 patients with RB were included in this study. This is illustrated in Figure 1.

Mean age at diagnosis was 17.2 months. The mean follow‐up time for all patients was 57.5 weeks (range seven to 263 weeks).

Four Group D eyes were found to have advanced disease and were subsequently referred for primary enucleation. Among the group undergoing primary enucleation, in the case of bilateral RB, the contralateral eye was classified as Group E in two patients and Group B in one patient whereas the other had unilateral Group D disease. The Group E eyes were treated with chemotherapy and subsequent enucleation. The normal eye received no treatment but was examined with EUA regularly. The mean age of diagnosis for patients who underwent primary enucleation of the Group D eye was 14.8 months. None of the patients treated with primary enucleation had any recurrences, metastases, or other secondary malignancies.

We attempted to preserve 15 Group D eyes (of 15 patients) with systemic chemoreduction, local consolidative therapy, and IViC. Patient parameters are summarized in Tables 1, 2.

The most common presenting sign was leukocoria (60%), followed by strabismus (20%). Other presenting features including proptosis amounted to 13.33% of the cohort. Bilateral disease was predominant in the study cohort with 14 (93.33%) bilateral cases, whereas unilateral disease was found in one (6.67%) patient. Of the bilateral cases, the majority consisted of Group E in the contralateral eye (85.72%) whereas Groups B and C were reported in one patient each (7.14%). The median age of diagnosis within this cohort was 32.0 months (mean: 34.7, range: 10.0-72.0). The mean follow-up time was 36.73±16.01 months (range: 17-71). A positive family history was found in two patients (13.33%).

**Table 1:** Demographic and clinical characteristics of patients with Group D retinoblastoma (RB)

Patient characteristics	Patients (n=15)	Percentage (%)
Gender		
Male	11	73
Female	4	27
Presenting signs		
Leukocoria	9	60
Strabismus	3	20
Both	1	6.67
Other	2	13.33
Laterality		
Unilateral	1	6.67
Bilateral	14	93.33
Classification of contralateral eye in bilateral cases (n=14)		
B	1	7.14
C	1	7.14
E	12	85.72
Family history of RB		
Present	2	13.33
Absent	13	86.67

**Table 2:** Age distribution in the cohort that underwent globe salvage

Variable	Median (Months)	Mean (Months)	Range (Months)
Age at diagnosis	32.0	34.7	10.0-72.0
Age at first symptoms	18.0	18.1	8.0-36.0

Overall, chemo-reduction with local consolidation successfully treated 11 of 15 eyes (73.33%). Before and after treatment, all eyes received laser therapy whereas five received cryotherapy in addition to laser therapy. One eye was given IViC in addition to local consolidative treatment. Of the eleven eyes that were salvaged, there were no side effects reported. Table 3 shows the treatment combinations offered.

**Table 3:** Treatment combinations offered IVC: Intravenous chemotherapy (vincristine, etoposide, carboplatin); IViC: Intravitreal chemotherapy (melphalan).

	Number of patients	Treatment modality
Primary enucleation	4	Upfront enucleation
Attempted globe salvage	15	
Successful globe salvage (n=11)		
	5	IVC + Laser + Cryotherapy
	5	IVC + Laser
	1	IVC + Laser + Cryotherapy + IViC (melphalan)
Treatment failure (n=4)		
	3	IVC + Laser + Cryotherapy + IViC (melphalan)
	1	IVC + Laser + Cryotherapy

**Figure 2:**
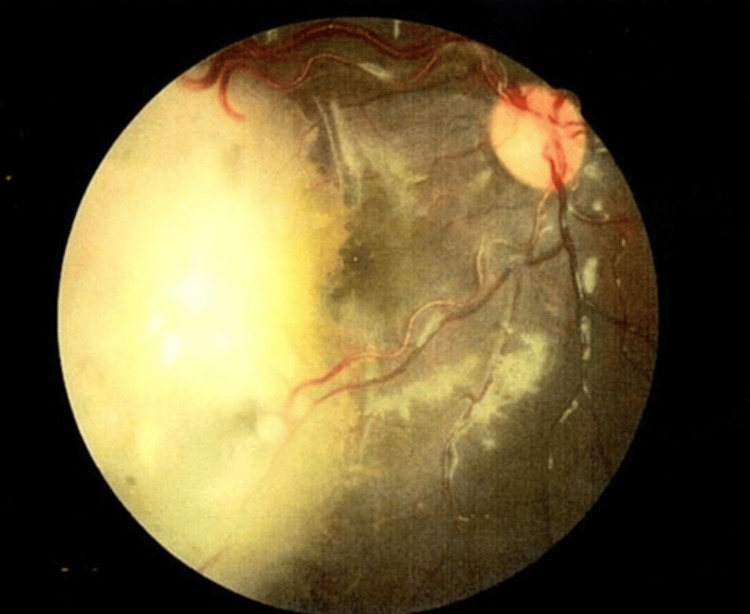
Right eye showing a large, elevated, exophytic mass involving half of the retina with diffuse seeds away from the tumor mass

**Figure 3:**
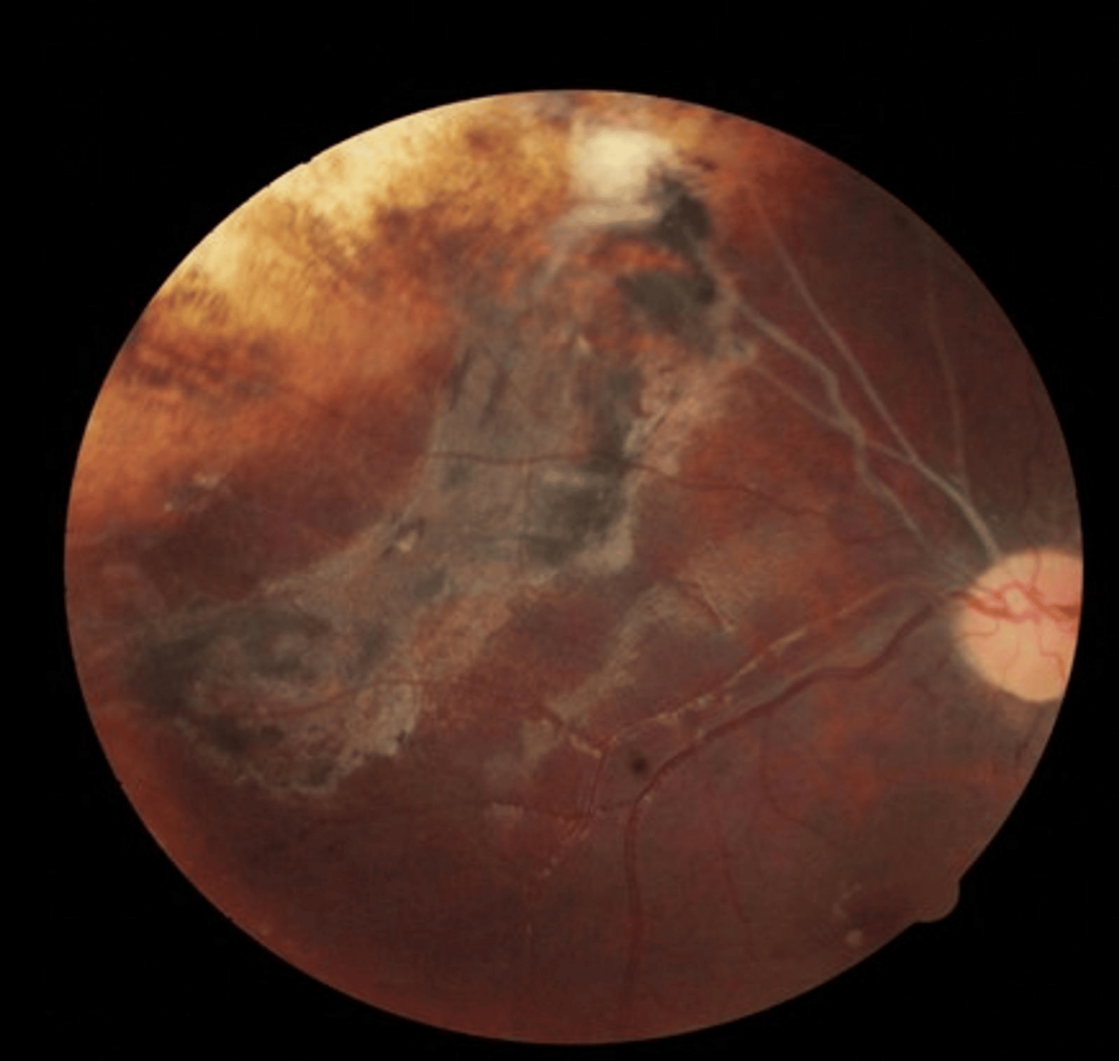
Same lesion at last follow-up with atrophic, inactive lesions, sclerosed vessels, disappearance of all seeds; multiple atrophic laser scar marks visible

Figures 2, 3 show the fundus photography of the right eye of a patient being successfully treated with IVC and local consolidative therapy.

**Figure 4:**
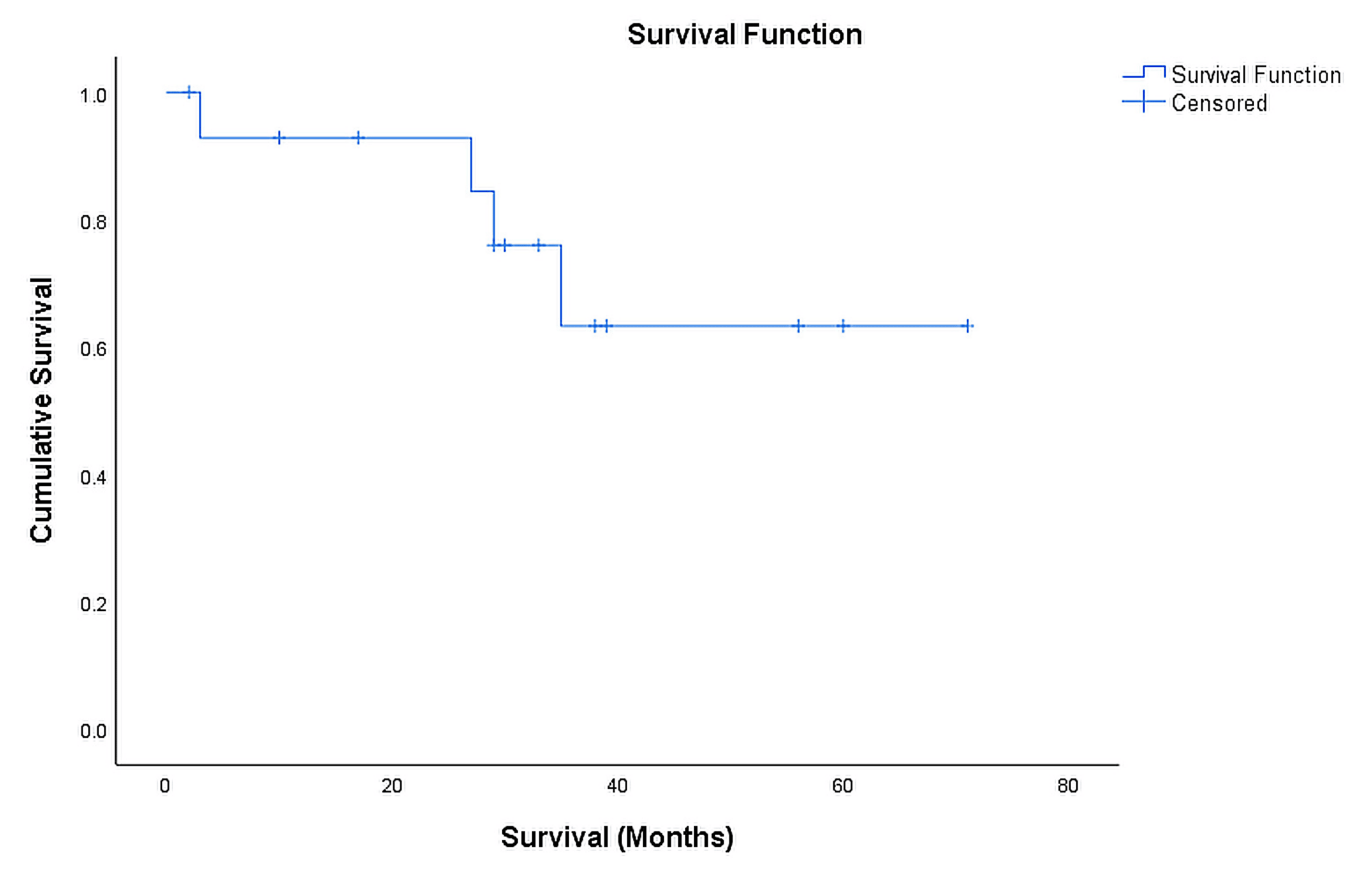
Kaplan-Meier estimates of globe salvage

Kaplan-Meier survival analysis showed an overall globe salvage rate of 93%, 76%, and 65% at one, two and three years, respectively. Figure 4 shows the cumulative eye survival according to Kaplan-Meier analysis.

Four eyes of four patients were secondarily enucleated. Of the four eyes that underwent secondary enucleation, two (50%) were performed in the first year of follow-up and two (50%) were performed during the third year of follow-up. Indications for secondary enucleation included recurrence of the tumor, extensive retinal detachment, phthisis after local consolidative therapy and presence of resistant, multiple vitreous seeds post-treatment. Three out of these four eyes received four IViC injections each but were found to have either persistent vitreous seeds or exudative retinal detachment on subsequent EUAs. All four enucleated eyes were sent for histopathological assessment and two of them were found to have high-risk histopathological features including optic nerve involvement in one eye and massive choroidal (>3 mm) invasion in the other.

## Discussion

The ICRB defines Group D eyes as having a significant tumor burden, including large tumors with diffuse subretinal and/or vitreous seeding. Salvage of Group D eyes is particularly challenging, and advanced cases are frequently managed with enucleation [8]. Pakistan contributes substantially to the RB burden in the Asia-Pacific region [9], yet resources for comprehensive management remain limited. This study examined outcomes of Group D RB treated at a center offering specialist multidisciplinary care. Such care is scarce in the region, and when combined with socioeconomic barriers, contributes to higher mortality compared with developed countries.

In our cohort, the most common presenting feature was leukocoria, followed by strabismus, both of which are well-documented presenting complaints of RB [10]. Genetic testing is rarely available in developing countries; therefore, family history was assessed verbally. Two patients (13.3%) reported a positive family history, suggesting that hereditary RB may be less prevalent than sporadic RB in this population, consistent with the findings of Kalsoom et al. [11].

The mean age of diagnosis in this study was 32 months, compared to 21 months in high-income countries such as the U.K., U.S., and France [12], and 20 months in Tehran [13], but similar to reports from India [14]. This delay in diagnosis is clinically significant: older age at presentation correlates with more advanced disease, lower salvage rates, and higher mortality. In our series, patients requiring enucleation were older than those whose eyes were salvaged. Many referrals were from rural areas, where early detection is nearly impossible, and financial constraints related to travel further delay presentation. These findings underscore the need for early detection initiatives and timely referral to specialist centers in Pakistan.

Of the 15 patients studied, 14 had bilateral intraocular RB, with most contralateral eyes classified as Group D or E. Only one patient presented with unilateral disease. Supawan et al. reported no significant association between high-risk pathological features and gender or laterality, consistent with our findings [15].

IVC combined with focal therapy remains the mainstay of treatment for Group D RB [16]. Despite emerging protocols, these eyes remain challenging to manage. The role of laser therapy is often underemphasized, yet it is an integral component of treatment. Laser is not only effective but also inexpensive and widely available in low-income countries [17]. Our outcomes highlight the importance of standardized laser therapy protocols, rather than relying solely on individual expertise, to improve reproducibility across centers, a point also emphasized by Soliman et al. [18].

Over a nine-year period, we attempted globe salvage in 15 Group D eyes, achieving an overall salvage rate of 73%. This outcome is comparable to findings from Fabian et al. in the U.K. [7]. Similar results have been reported in India, where Kaliki et al. achieved a salvage rate of 58% with systemic chemotherapy [19], and Khaqan et al. reported 72.7% with IVC, laser therapy, cryotherapy, and IViC [20]. Tan et al. also reported high salvage rates (87%) in China using systemic chemotherapy with local consolidation [21]. Collectively, these studies validate our findings and support IVC with focal therapy as an effective treatment strategy for Group D RB.

Of the four patients treated with IViC in addition to systemic chemotherapy, three eventually required secondary enucleation. This aligns with Rehman et al., who found no significant additional benefit from IViC [22]. By contrast, Amin et al. reported more favorable results, which may reflect differences in sample size or vitreous seed morphology [23]. Our findings suggest that large cotton ball-like seeds and those associated with persistent tumor masses or exudative retinal detachment are more resistant to IViC, frequently leading to enucleation [24].

While several international studies have reported salvage outcomes for Group D RB, few have been conducted in Pakistan. Variability across studies may reflect differences in classification systems, underscoring the importance of clearly stating the criteria used [25]. Although intra-arterial chemotherapy has demonstrated superior salvage rates compared to IVC in high-income countries [26], our results demonstrate that comparable outcomes can be achieved in low-resource settings when specialist centers and multidisciplinary care are available.

Limitations

This study has several limitations. First, patients undergoing primary enucleation were excluded, which may have introduced selection bias. Second, visual acuity outcomes before and after treatment were not assessed, partly due to incomplete records. Third, follow-up was truncated in some cases because of incomplete contact information and missed appointments. Finally, the relatively small cohort size limits the generalizability of our findings. Nevertheless, with a salvage rate of 73% and no recorded deaths or metastatic disease during the study period, this study demonstrates encouraging outcomes and provides evidence to support effective RB management in Pakistan.

## Conclusions

This study demonstrates that IVC combined with local therapies such as laser photocoagulation and cryotherapy remains a highly effective treatment option for Group D RB in resource-limited settings. Despite the advancements in intra-arterial chemotherapy, IVC has proven to achieve significant globe salvage rates, with 73% of eyes salvaged in this cohort. These findings are particularly important for developing countries, where socioeconomic barriers and limited access to advanced technologies often hinder optimal treatment outcomes.

The results underscore the importance of early diagnosis and referral to specialist centers to improve treatment success rates. Furthermore, the study highlights the need for standardized training programs in local consolidative therapies, such as laser, to enhance reproducibility and effectiveness across healthcare providers. While IViC showed limited success in cases with resistant vitreous seeds, further research is needed to optimize its use and understand its role in conjunction with IVC in these challenging cases. The study’s findings emphasize the potential for multidisciplinary care to address the unique challenges of managing advanced RB in LMICs, paving the way for better patient outcomes and reduced disease burden.
